# Management of Traumatic Hyphema and Prevention of Its Complications

**DOI:** 10.7759/cureus.15771

**Published:** 2021-06-20

**Authors:** Evan J Chen, Airaj Fasiuddin

**Affiliations:** 1 Ophthalmology, University of Central Florida College of Medicine, Orlando, USA; 2 Pediatric Ophthalmology, Nemours Children’s Hospital, Orlando, USA

**Keywords:** hyphema, glaucoma, pediatric cataract

## Abstract

Hyphema is defined as an accumulation of blood in the anterior chamber of the eye. Numerous conditions can lead to the development of hyphema, with blunt and penetrating trauma serving as the most common etiologies. Although the annual incidence of hyphema is relatively low, this condition must be recognized timely in order to manage and prevent its complications, such as glaucoma and corneal blood staining. This case report presents a 17-year-old adolescent who developed a hyphema complicated by a transient elevation in intraocular pressure following a high-speed motor vehicle accident. She responded to medical treatment and showed no signs of glaucomatous optic nerve damage at the end of her treatment course. The pathophysiology, clinical signs and symptoms, complications, medical and surgical treatments, and prognosis of hyphema are subsequently discussed.

## Introduction

Hyphema is defined as a collection of blood in the anterior chamber of the eye. Microhyphema is a small amount of blood that is visible only under microscopic examination. Hyphema is most commonly caused by blunt or penetrating trauma. Additional causes of hyphema include neoplasm, uveitis, juvenile xanthogranuloma, coagulopathies, and iatrogenic after intraocular surgery [1,2]. Even small amounts of blood within the anterior chamber can lead to complications such as glaucoma, corneal blood staining, and secondary hemorrhage; thus, proper management of this condition is crucial to prevent loss of vision. This report presents a case of an adolescent who developed a traumatic hyphema. It aims to discuss the pathophysiology, management, and complications of hyphema, as well as the prevention of its complications.

## Case presentation

A 17-year-old female with no past medical history was brought to the emergency department (ED) after a high-speed motor vehicle crash. Initial Glasgow Coma Score was <4 on the field and was 7 upon arrival to the ED. The initial ophthalmic presentation included periorbital edema and ecchymosis bilaterally, left eyelid laceration, fixed and dilated left pupil, and multiple facial lacerations and edema. Computed tomography (CT) of the head revealed the absence of acute intracranial damage, optic nerve injury, proptosis, or globe damage. Maxillofacial CT found multiple facial bone fractures. Hyphema of the left eye was also identified by an ophthalmologist and treated with one week of 1% topical prednisolone. The hospital course was without any complications. She was discharged eight days later per family request.

The patient presented to a second ophthalmologist three weeks later for left eye pain, persistent blurry vision, and photophobia. Visual acuity was 20/20 in the right eye (OD) and 20/150 in the left eye (OS). Visual fields were full to finger count bilaterally. Tonometry revealed intraocular pressures (IOPs) of 18 mmHg OD and 24 mmHg OS. The right pupil was reactive to light, and the left pupil was enlarged at 7 mm with an abnormal response to light. Both eyes demonstrated intact alignment and extraocular movements. All examination findings in the right eye were within normal limits. Slit lamp examination of the left eye revealed 2+ mixed cells (inflammatory cells and erythrocytes) within the anterior chamber without visible layering of blood, consistent with grade 0 hyphema (microhyphema). Trace diffuse injection of the conjunctiva and sclera was also observed. Iris sphincter muscle tear was visible, which caused the increased left pupil size. Examination of the lens revealed a large, 3+ posterior subcapsular cataract with mild fibrosis of the anterior lens capsule. Vitreous was prolapsed anterior to the lens superonasally. Eyelids and cornea were intact. Funduscopic examination of the left eye revealed normal retina without tears or detachment, an intact optic disc, and optic nerve with a cup-to-disc ratio of 0.5 (C/D = 0.5 OD). Based on these findings, diagnoses of microhyphema and traumatic cataract of the left eye were made. The patient was prescribed topical prednisolone 1% and atropine 1% twice daily for the treatment of microhyphema, and topical timolol 0.5% twice daily for elevated IOP of the left eye (normal IOP 8-22 mmHg). She was monitored closely for the development of glaucoma secondary to hyphema or trauma. Past medical, surgical, family, and social histories were unremarkable.

Subsequent evaluations revealed subjective improvement of ocular pain and photophobia without improvement of visual acuity in the left eye. Visual acuity of the left eye deteriorated from 20/150 at three weeks status post-injury to 20/300 12 weeks status post-injury. The left pupil remained large with an irregular response to light. IOPs increased from 24 to 27 mmHg at nine weeks status post-injury; thus, timolol was replaced with topical Cosopt (dorzolamide-timolol) 22.3-6.8 mg/mL twice daily. At the most recent follow-up (12 weeks status post-injury), the patient’s IOP improved to 17v mmHg. Slit lamp findings of the iris, lens, and vitreous remained the same as previously noted, but conjunctiva and sclera were now white and quiet, and the anterior chamber revealed only trace amounts of mixed cells at nine and 12 weeks status post-injury. Funduscopic examination remained stable with no change in optic nerve appearance to suggest glaucomatous damage. Prednisolone was tapered off and atropine was discontinued at nine weeks status post-injury due to improvement of microhyphema. The patient was advised of a potential increase in IOP as hyphema resolves, and of potential development of angle recession glaucoma following the trauma. Care was then transferred to an adult ophthalmologist for monitoring of IOP and eventual cataract extraction.

## Discussion

The annual incidence rate of hyphema diagnosed in ED visits is approximately 0.52/100,000, with young males accounting for the majority of cases [2]. Ocular trauma leads to tear of blood vessels in the anterior chamber and injury to the iris, ciliary body, and their associated vasculatures. Disruption of vessels such as the recurrent choroidal arteries and major arterial circle of the iris allows for the accumulation of blood within the anterior chamber, resulting in hyphema [1,3].

With the development of hyphema, patients may complain of blurred vision or complete loss of vision, and pain and photophobia typically proportional to the amount of blood within the anterior chamber. As with this patient, symptoms of eye pain may occur due to elevated IOP, a complication of hyphema. Diagnosis of hyphema is often confirmed by a slit lamp examination, and grading of hyphemas is crucial in guiding management and determining prognosis.

An elevated IOP due to blockage of the trabecular outflow by red blood cells and inflammatory debris is the most common complication of hyphema [1]. Although all grades of hyphema, even a microhyphema, can cause such a complication, the likelihood of developing glaucoma from hyphema is typically directly correlated to a high grade of hyphema [1]. It is crucial to note that patients with sickle cell disease or trait are more susceptible to developing secondary glaucoma because of increased blockage of the trabecular outflow by sickled cells, and thus have a higher likelihood of visual loss from hyphema, regardless of its severity [4]. Therefore, screening for sickle cell disease or trait is recommended for African American patients with hyphema [4].

Other complications of hyphema include peripheral anterior synechiae formation, angle recession, secondary hemorrhage, corneal blood staining, and amblyopia in children [1,5]. The risk of these complications is significantly increased in patients who rebleed, which occurs in up to 38% of patients with hyphema, typically within the first week after the initial injury [1,3,6].

Management of hyphema ranges from medical to surgical and aims to eliminate any potential complications as discussed above. Most hyphema are self-limited and usually resolve within one week; bed rest, head of the bed elevation more than 30 degrees, and eye shields may be sufficient. Bed rest or activity restriction prevents the risk of rebleeding. Strict bed rest is encouraged in patients susceptible to rebleeds, such as those with sickle cell disease, coagulopathies, or severe hyphema. Elevation of the head of the bed enables blood to pool at the bottom of the chamber thereby improving vision and prevents obstruction of the trabecular outflow by cells. Wearing an eye shield is also encouraged to protect the eye. Anticoagulants, antiplatelets, and analgesic medications such as nonsteroidal anti-inflammatory drugs and aspirin should also be discontinued for the prevention of continued or secondary hemorrhage. Medical treatments include the use of topical corticosteroids, cycloplegics, antifibrinolytics, and antiglaucoma drugs. Of which, steroids and cycloplegics are used most frequently. Corticosteroids are useful in the prevention of secondary hemorrhage and formation of peripheral anterior synechiae, as well as reduction of inflammation [7]. Although they do not significantly prevent the risk of secondary hemorrhage, cycloplegics such as atropine, are also commonly used to reduce the risk of posterior synechiae formation and to improve patient comfort by preventing spasms of ciliary muscles [7]. Antifibrinolytics such as tranexamic acid and aminocaproic acid also reduce the risk of secondary hemorrhage by promoting coagulation [1,7,8]. Finally, beta-blockers, alpha agonists, and carbonic anhydrase inhibitors are useful in reducing IOP.

Surgical management is indicated in cases refractory to medical treatment, persistent high-grade hyphemas, corneal blood staining, persistently elevated IOP, and uncontrolled glaucoma [1,3]. A lower threshold for surgical treatment is placed in patients with sickle cell disease or trait with persistently elevated IOP for more than 24 hours [1,3]. Surgical options include anterior chamber washout with irrigation and aspiration, trabeculectomy, peripheral iridectomy, and anterior chamber paracentesis [1,3]. However, all surgical options are associated with risks of secondary hemorrhage and damage to the cornea, lens, and iris, and are considered only in cases not responsive to medical treatment [3].

## Conclusions

Although most hyphemas are self-limiting, adequate monitoring of complications and visual acuity is crucial. Associated complications such as cataract and elevated IOP are common findings. Glaucomatous vision loss can be prevented with close monitoring and timely medical treatment. In the context of this patient, a traumatic cataract will likely hinder her from recovering her visual acuity, and long-term follow-up with an ophthalmologist for continuous monitoring of complications is highly recommended.
